# Nurse assistants’ perception of caring for older persons who are dying in their own home: An interview study

**DOI:** 10.1186/s12904-024-01399-2

**Published:** 2024-03-12

**Authors:** Magdalena Annersten Gershater, Josefin Brenner, Malin Nordberg, Ami Hommel

**Affiliations:** 1 Department of Care Science, Faculty of Health and Society, Malmö University, Jan Waldenströms gata 25, Malmö, 206 05 Sweden; 2 Department of Health and Social Care, Home Care Kungsparken, Malmö Municipality, Västra Kanalgatan 4, Malmö, 211 41 Sweden; 3 Department of Health and Social Care, Malmö Municipality, Villa Vikhem, Vikhems bygata 100, Staffanstorp, 245 46 Sweden

**Keywords:** Home care, Interview study, Nurse assistant, Older person, Palliative care

## Abstract

**Background:**

As the proportion of older persons in society increases, there is a growing trend towards providing end-of-life care in their homes. Palliative care is a complex and knowledge-demanding form of care, and nurse assistants are those who work closest to the older person at the end-of-life in their own homes. However, nurse assistants sometimes have low educational and insufficient levels of knowledge in palliative care, which can affect the quality of care they provide. Moreover, nurse assistants’ experiences are relatively unexplored in this context. The purpose of the study was to illuminate nurse assistants’ experiences in caring for dying older persons at home.

**Method:**

An empirical, qualitative interview study was conducted with 14 nurse assistants with experience of palliative care in homecare. The material was analyzed using thematic content analysis.

**Results:**

From the nurse assistant’s experiences, one main theme emerged: doing everything possible for the dying older person despite challenges. Moreover, three sub-themes emerged: making a difference at a crucial time, death awakens emotions, and balancing personal and professional relationships. The nurse assistants’ saw their role primarily as relieving symptoms but also focusing on next of kin. The following are described as essential parts of their role: carrying out practical nursing tasks, focusing on the physical environment, working alone and seeking help from colleagues due to a physical distance to the other members of the multidisciplinary team. The nurse assistants experienced a lack of support as there was no structured guidance or debriefing available in difficult emotional situations. Furthermore, they disclosed that they were left alone to deal with their feelings.

**Conclusion:**

This study demonstrates that nurse assistants strive to provide comprehensive care for dying older persons despite facing obstacles from their working conditions and work organization. They lack supervision and education in palliative care, but they rely on their experience-based knowledge to a large extent and provide care according to the four cornerstones of palliative care.

**Supplementary Information:**

The online version contains supplementary material available at 10.1186/s12904-024-01399-2.

## Background

The number of older persons worldwide is increasing rapidly, with its population projected to rise from 10% in 2022 to 16% in 2050 [1]. According to the World Health Organization (WHO) [2], an estimated 56.8 million people, including 25.7 million in the last year of life, need palliative care annually. In Sweden, the proportion of older persons has been increasing since 1970. In 2022, the number of older persons aged 65 and above was 20% of the population, which is 2.1 million out of a total of 10.5 million inhabitants [3].

Most of the older population spend their last year at home, with many wishing to die in the company of their next of kin [4, 5]. Supporting such persons at the end-of-life to enable them to die at home is a significant task for the nurse assistants (NA) and the district nurses (DN) [6]. In addition, older people are often dying of multiple morbidities, which makes it challenging to manage their symptoms. Consequently, there is an increasing need for home-based palliative care (HBPC) to provide ongoing symptom management.

HBPC is a team-based person-centered model of care for persons and their next of kin affected by severe illness. The team are providing palliative care in patients’ homes in collaboration with primary care [7]. End-of-life care refers to the last phase of PC, which is given during the last weeks and days of life PC decision should be made in consultation with the patient [8]. The DN and NA should provide physical, psychological, social, or existential needs.

End-of-life situations for an old dying person often show a multifaceted picture of the complexity of palliative care. The health condition can change with short-term improvements, but a slow deterioration is inevitable. Therefore, collaboration in multi-professional healthcare teams is necessary to provide good palliative care and meet a dying person’s needs. Healthcare providers provide general palliative care with basic knowledge and expertise in palliative care. However, if a person has complex symptoms or whose life situation entails special requirements, a multi-professional team provides specialist palliative care with specific knowledge and expertise in palliative care. This care is administered in a specialist palliative organization or within a unit providing general palliative care with the support of a palliative care team [9].

Good palliative care is based on the four cornerstones of symptom relief: multi-professional collaboration, communication, relationships, and support for related persons [9]. These cornerstones can support the healthcare providers and be used to quality assure the palliative care in the home to promote the dying persons’ opportunities for participation in their care and to facilitate as good a life as possible at the end-of-life [10].

The limited number of studies presented below carried out with NAs in municipal care do not seem to focus on the NAs’ role in the specific relief of symptoms. However, several studies from different parts of the world show that there is a need for improvement for healthcare providers to be able to deliver good palliative care. One study from China shows that NAs’ attitudes to palliative care are moderate and that they need more knowledge [11] to promote high quality palliative care. A Swedish study [12] identified healthcare providers’ need for managing existential aspects of the ageing body, life and death, and engaging in existential conversations when appropriate. One Swedish study [13] highlighted that young healthcare workers without the education and experience of a dying person might be vulnerable in palliative care. In another Swedish study [14], the NAs emphasized that they needed supportive and experienced leadership. A study from the USA [7] discussed challenges with patient referrals, specifically, lack of palliative care knowledge in both providers and patients and their families and poor communication with patients referred to HBPC. Thus, palliative care educational interventions are needed. A review article [15] highlighted that the role of healthcare assistants both is hidden and undervalued. Bolt et al. (2020) showed that nursing staff with different education levels in the Netherlands endorse similar support needs, including recognizing and managing pain. Lacking skills and knowledge may adversely affect the quality of palliative care [16]. In another Swedish study [17], the availability of RNs in nursing homes was identified as a critical factor in providing good care at the end-of-life.

The goal for all healthcare organizations in Sweden is to provide care on equal terms, regardless of age or whether the care is provided in a hospital or the individual’s home [18]. NAs are key staff in providing palliative care because they spend more time with patients than other healthcare professionals, because they are first to recognize changes in the dying persons’ physical and cognitive functioning [19], and because they report to the RNs [20].

Therefore, this study aimed to illuminate NAs’ experiences providing end-of-life home care for the dying older person.

## Method

### Study design

A qualitative interview study was conducted using the semi-structured interview method [21]. The study applied a qualitative content analysis with an inductive design [22] to investigate NAs’ individual experiences. The study’s results were contributed to by context and relationships with dying older persons, their next of kin, and other healthcare providers.

### Settings

In Sweden, older people who live in their own homes and require support in daily living activities can receive care from NAs through the municipalities’ social work organizations [23, 24]. The healthcare team in Sweden includes a registered nurse (RN) (often a DN or an RN specialized in older care) who is responsible for the general PC care, with medical support from a general practitioner (GP), while NAs perform most nursing interventions. A high school degree or equivalent is required to become an NA.

The study was conducted in urban general home healthcare in a municipality, in the south of Sweden, in the spring of 2022.

### Sample

The inclusion criteria were at least one year of work experience in an older person’s home and experience in palliative care for older people in this context. The managers of six home healthcare units gave informed consent to the study. In total, 14 NAs from two home healthcare units responded. The two home healthcare units’ managers acted as gatekeepers and provided information to the NAs. Twelve women and two men (age 25–62 years, median 62 years, with working experience 1–37 years, median 15 years) agreed to participate in the study and contacted the authors JB and MN to schedule an interview.

### Data collection

All face-to face interviews were conducted during working hours at the NAs units, in a comfortable and setting. A semi-structed interview guide with open questions was used to answer the aim of the study and to ensure that the four cornerstones were included. For example, the interviews were opened with the question: “Tell me about your experience of caring for older people at the end-of-life in their home?. To ensure the quality of the interview guide, a pilot interview was conducted. No changes were made to the interview guide after the pilot interview, and the pilot interview was included in the study’s results. The interviews lasted 35 to 74 minutes. To reduce the risk of bias that may develop if only one person conducts all the data collection, the authors JB and MN conducted the first ten interviews, while the remaining four were conducted with one author, either JB or MN. Each interview was recorded and transcribed verbatim.

### Data analysis

The authors used qualitative thematic content analysis with an inductive approach as proposed by Burnard [23] (Table 1). Content analysis was a suitable method for extracting meaningful content from the NAs’ experience of caring for older persons in end-of-life situations. JB and MN first listened and then read the transcribed interviews repeatedly and individually to obtain an overall understanding of the material. Next, they highlighted individual text related to the aim of the study. In the second step, the highlighted parts were condensed. In step 3, the remaining material was then jointly categorized, and codes with similar meanings were arranged and compared to ensure consensus. Next, codes were grouped and regrouped into general themes (i.e., thematic categorization). The results were compiled into themes and subthemes after repeated comparisons and reductions of the categories. Finally, the distinctive characteristics of each theme were identified and described to identify constructs until the final themes integrating all NAs’ perspectives were formed. See Table 2. To ensure credibility, additional material processing was performed to determine if the themes and subthemes reflected the original text. To ensure trustworthiness, JB and MN critically discussed all the steps in the analysis and the emerging themes and subthemes with MAG and AH until a consensus was reached.

**Table 1:** Subthemes and theme

Subthemes	Themes
Making a difference at a crucial time	Palliative care in the old person’s own home involves doing everything possible for the dying older
Death evokes emotions	
Balancing personal and professional relationships	

**Table 2:** Qualitative thematic content analysis with an inductive approach as proposed by Burnard

Step 1 JB and MN first listened and then read the transcribed interviews repeatedly and individually to obtain an overall understanding of the material	Step 2 In the second step, the highlighted parts were condensed. Next, they highlighted individual text related to the aim of the study	In step 3, the remaining material was then jointly categorized, and codes with similar meanings were arranged and compared to ensure consensus. Next, codes were grouped and regrouped into general themes (i.e., thematic categorization). The results were compiled into themes and subthemes after repeated comparisons and reductions of the categories. Finally, the distinctive characteristics of each theme were identified and described to identify constructs until the final themes integrating all NAs’ perspectives were formed

## Results

The theme ‘Palliative care in the old person’s own home involves doing everything possible for the dying older person’ expressed the NAs’ commitment to doing all they could to ensure the dying older person would be as well as possible at the end-of-life. As a result, it became natural for the NAs to go beyond the professional role and invest a private part of themselves in the relationship. The theme is revealed from three subthemes: (1) making a difference at a crucial time, (2) death evokes motions, and (3) balancing personal and professional relationships.

### Making a difference at a crucial time

The NAs’ described that they could make a significant difference for the dying older person and their next of kin by seeing the overall picture and alleviating symptoms. The NAs’ emphasized that they tried to do everything possible to offer safety and to fulfil their patient’s last wishes. They felt that they made a valuable difference to the person at the end-of-life through being flexible, sensing the mood in the room, lowering their voice, and not revealing any stress when caring for the dying person’s physical well-being, for example, by performing oral care, changing incontinence pads, and performing position change. Many NAs considered the following as crucial in end-of-life care: talking to the dying older person even when they could no longer communicate but could still hear and ensuring they were not left alone.

The NAs described enabling their patients to preserve their personalities at the end-of-life by helping them live as usual for as long as possible, despite the focus on palliative care. The NAs also had to focus on the next of kin by being available for conversation, answering questions, or giving a hug. It was important that the next of kin were satisfied with the care so that they could relax and rest.

The NAs actively observed the dying person’s movement patterns, sounds, or facial expressions to ascertain if they needed some sort of relief. For example, they could relieve anxiety by having calming conversations or by responding calmly. Sometimes they would try to relieve anxiety through diversionary conversations or through using a sense of humor to lighten the mood. The NAs also experienced that holding their hand, patting their cheek, or massaging their feet could relieve pain and anxiety:

Sometimes you must hold their hand and talk to them if they are conscious. What have they done in their life? Some are so worried about death; however, some are relaxed. So, try in some way so they do not think about that. Try to engage them in conversation. (NA1)

When the NAs could not independently provide relief to the dying older person, they concluded that they needed symptom relief through an injection from an RN. For some NAs, contacting an RN was the first thing they did when the dying person showed pain or anxiety symptoms. In contrast, other NAs tried to provide relief with non-pharmacological alternatives such as position changes, help to the toilet, massage before contacting the registered nurse.

### Death evokes emotions

The NAs’ expressed how death and working close to a dying older person evoked feelings that could be experienced as both valuable and challenging. Having a professional role in end-of-life palliative care often involves having a personal relationship with the person and experiencing sadness. Conversations about death with the dying older person were a natural part of the work for some NAs, and they emphasized the importance of being open and receptive to such conversations. Other NAs described how they sometimes avoided conversations about death by changing the subject. The NAs did not initiate conversations about death, but they responded to them on the initiative of the older person. The older person often expressed their thoughts about how they wished their remaining time in life to be and how they would like to be cared for after death:

We knew her before she deteriorated and became palliative, making it more manageable. We knew what she liked and did not like, and how her routines worked. So, we only needed a little help from relatives. Moreover, she thought that was great, “Oh, so good that you know.” Furthermore, she was satisfied and said, “That is how I want it; that is good”. (NA 9)

The NAs found the dying process, with severe symptoms and suffering, complex to respond to and handle, even though an older person’s death is considered a natural part of life. They communicated that when an older person in palliative care dies the death was perceived as peaceful and a relief for that person, who no longer must suffer. When a person requires end-of-life palliative home care, the NAs revealed they accepted that death was imminent and that they could manage to deal with the emotions that naturally arose. However, if the death occurred suddenly, for instance, if they unexpectedly found a deceased person at the home, this would be more challenging to deal with emotionally.

The interaction with the next of kin could lead to challenging situations for the NAs. For example, there were occasions when relatives did not accept that their old family member would die or who did not understand what end-of-life palliative care entails. Such situations could lead to challenging questions from the next of kin – questions that the NAs had difficulties answering. Thus, they had to repeatedly inform the next of kin about what constitutes palliative care. The NAs handled these relatives using different strategies, such as answering to the best of their ability, referring to the RN for support, or avoiding answering.

Although complicated feelings could arise when providing palliative care for older people at home, the NAs, nevertheless, considered it both essential and rewarding work. They expressed that they were making a difference to the dying older person at a particularly significant time. Moreover, sitting with a dying person could arouse thoughts about life and death and make them think about and value their own life more. The NA participants felt good about doing their best to help the older person and, in return, they received validation for their work through appreciation from both the dying older person and their next of kin.

### Balancing personal and professional relationships

The boundaries between the professional and the personal relationship the NAs build with their older dying person were expressed as challenging to balance. At times these boundaries became blurred, and the relationship was often more personal than professional. However, the NAs, generally, did not perceive it as harmful to be more personal than professional. Indeed, the NAs testified to how meaningful this relationship was in their work situation.

They expressed how they gladly went beyond the requirements of their professional role to make a dying older person feel better. Many of their relationships with the older persons had been built over time and included continuity of care, which better facilitated their work as they had in-depth knowledge of the patient’s preferences. At the same time, the NAs emphasized that also the person they did not know before received good care.

Different emotions were evoked upon the passing of a familiar older person. Some NAs felt sad but acknowledged this was part of the work and not challenging to deal with; they tried to set aside their feelings and act professionally for the peers. However, others mourned the older person and described it as losing a family member, thus explaining why it was necessary for them to say goodbye before death and be present until the end. According to the NAs, attending the funeral was valuable as it enabled them to deal with the grief and loss of the older person with whom they had built a relationship, sometimes over several years.

The NAs often spoke of physical distance, both being distanced from their home-care colleagues and, above all, from the other team members. Because they usually work alone, they had to call colleagues when in need of help. Professional groups collaborating in the palliative home-care team around the dying older person are the RN, the physiotherapist, and the occupational therapist. However, collaboration was almost exclusively with the RN for end-of-life palliative home care.

The NAs related needing emotional and practical support from the multi-professional team in complex, challenging situations. In such emotive situations, their NA peers provided most support, while the RNs provided good practical support. Good accessibility to the RNs was a decisive factor for a smooth collaboration around the dying older person, with communication mainly taking place by phone due to the physical distance between their respective workplaces. Some NAs said they only called the RN when they felt there was an important issue but were sometimes not acknowledged, which can destroy the sense of trust and cooperation if the RN is unable to get to the dying person as quickly as needed. Further, the NAs found it frustrating when the RN only focused on pain relief without addressing the older adult’s other needs. At the same time, the NAs’ descriptions of collaboration with the RN focused on contact for symptom relief in injection form.

The NAs underlined that they require flexibility and much time to perform palliative care with good quality, but that the lack of time can be an obstacle to performing their duties well. At the same time, being flexible with how their duties are prioritized could create more time for what is most important to the dying older person:

The tricky thing with such palliative care at home is the time. It is impossible […] No, but we have calculated how long a hygiene visit takes or how much a shower takes. It is impossible to think about when you come across someone very sick because that person might not be able to handle us doing those things right then because of pain or something. So, you have to try to rearrange the schedule. (NA 8)

Caring for dying persons in their own homes could sometimes entail specific difficulties. For example, the NAs might perceive the dying older person as lonely and the did not know how to deal with this. Further, they believed that the quality of palliative care was negatively affected when colleagues lacked knowledge of palliative care or did not have sufficient language skills to respond appropriately to the dying person and their next of kin. The NAs also experienced a lack of support as there was no structured guidance or debriefing available in difficult emotional situations, and they felt that they were left alone to deal with their feelings. As a result, the NAs continued to work through the incident without talking about it, except with their closest colleagues:

What worries me the most is that many people who work in home care have no education; they know nothing about diseases, they know nothing about dementia, and they know nothing about palliative care. (NA 3)

## Discussion

The aim of this study was to illuminate NAs’ experience of providing end-of-life home care for the dying older person. The findings showed that palliative care in the older person’s home involves doing everything possible for the dying older person. The NAs, who tended to have the least education, had to face death and the courses of dying, for which they needed recognition and support. Moreover, they were the first to recognize symptom changes. Further, they provided palliative care based on the four cornerstones of symptom relief: multi-professional collaboration, communication, relationships, and support for related persons [9]. The four cornerstones served as support for the NAs to promote the next of kin and the dying persons’ opportunities for participation in the care. The NAs’ primarily saw their role as helping with physical needs and relieving symptoms of anxiety and pain, which they did by having calming conversations and physical contact, as well as calling the RN when injections where needed. Which is a good example of how the NAs where able to build a good relationship, communication, and multi-professional collaboration.

Most people prefer to die at home [25]. Therefore, the NAs expressed that they try to do everything possible for the dying person to the best of their ability. They wanted to support the related persons and wished to make a difference in the lives of dying persons and their families. The NAs they value having a holistic view of the dying older person. However, death arouses difficult emotions, and unexpected deaths cause complicated feelings. Despite these challenges, NAs try to do everything for the dying person to the best of their ability and adapt their whole way of being when they enter a home where an older person is dying, showing respect for the fragile situation that the person and their next of kin are in. The same result has been described by Beck et al. [14] about palliative care in nursing homes, where end-of-life palliative care contrasted with daily care. This finding has been confirmed by another study, which also discussed how the pace slowed, and the nurses’ attention was directed to the person’s need for presence and touch [26]. There seemed to be a sharp line between daily care and end-of-life palliative care, although the older person had been frail for a long time and had life-threatening illnesses requiring a palliative approach [14].

In the present study, palliative care included the last weeks or days before death. However, palliative care also includes the early phase when the person with a chronic or incurable disease receives life-prolonging treatment. Nevertheless, previous studies have also shown that personnel working with older people often view palliative care as limited to the short time at the end-of-life [14, 27]. This is because the dying process for the older person can be protracted [28], and it can be difficult to identify signs that death is approaching. Furthermore, the extent to which the RNs and NAs receive information about the health status of the older persons in their care is still being determined. Nonetheless, personnel working with older people can often identify early signs of dying, which could contribute to the older persons and their relatives having the opportunity to prepare for and plan for the last moments [27, 29].

The results of this study indicate that the NAs try to relieve physical and psychological symptoms. This study confirms the results from other assisted living facilities describing how to healthcare personnel try to provide good care, for example by ensuring the older person’s well-being by moistening the mouth, preventing pressure ulcers, or being close and available [26, 29]. The limited number of studies carried out with NAs in municipal care do not seem to focus on the NAs’ role in the specific relief of symptoms, which is surprising because they constitute the category of personnel in end-of-life palliative home care for older people who spend the most time with the dying person. Prevention and early symptom detection are essential to optimally relieve symptoms [9] optimally. Our results – the NAs describing how they relieve pain and anxiety, how death evokes emotions, and the boundaries between the professional and personal relationship – highlight the importance of having experience in palliative home care to give dying older persons the best opportunity for effective symptom relief.

Caring for a dying person’s existential requirements is crucial in good end-of-life palliative care [9]. In this study, the NAs often dealt with conversations of an existential nature. However, they tended to adopt an avoidant attitude towards these conversations. Avoiding talking about death has also been reported in other studies carried out in nursing homes [14, 26, 30]. Based on the results of the present study, the ability to respond to existential questions depends on the NA’s personality and work experience and not on any previous education. Therefore, those who work with end-of-life palliative care must be provided with the opportunity to discuss ethical issues, receive training on existential needs, and how to meet them [14, 26, 31]. These measures would increase the possibilities for high-quality end-of-life palliative care for more older people.

The cornerstones good communication and relationships are essential for promoting a dying person’s quality of life. But based on the results of this study, these factors also appear necessary for nursing personnel. The NAs emphasized the balance between professional and personal relationships and the value of knowing the older persons in their care. This result was confirmed in the study by Beck et al. [32], where the nurses valued the relationship with older people and did more than needed. Working in someone’s private home, meeting the person regularly for an extended period, and becoming like “one of the family” can easily blur the boundaries between the professional and personal [33, 34]. Becoming emotionally involved can be seen as an inevitable and a welcomed part of the work. However, it also risks going beyond the healthcare provider’s capacity because the high workload and ethical conflicts may compromise the limits of one’s professionalism. The relationship with the dying older person can also motivate the caregiver in their work if the relationship involves mutual appreciation [33]. All healthcare personnel working in the care of older people must learn how to provide end-of-life care and be able to support both the dying persons and their next of kin. Nonetheless, there is a shortage of personnel with appropriate educational competency from high school and work experience [24]. Furthermore, according to the Swedish National Board of Health and Welfare [9], the municipalities have neglected education and counselling for personnel in end-of-life palliative care; this is considered fundamental for safeguarding people at the end-of-life, providing necessary support and alleviating their symptoms.

Meetings with relatives could lead to difficult situations and emotions. The NAs are usually the ones who have the most contact with the patient’s next of kin. In this study, the NAs saw their knowledge as insufficient to adequately answer questions and respond to the next of kin who did not understand palliative care. In such cases, the NAs sought help from the RN. Next of kin only sometimes receive sufficient information about the end-of-life situation, thus making them poorly prepared for the final moments of their loved one’s life [35]. Moreover, because the NAs cannot access complete medical information or provide full information about the older dying person’s situation, they need professional support. Although they can cope independently in difficult situations, they also need the employer’s support in and reflection on handling such challenging situations. The NAs believed that both they and the RN collectively formed a sufficiently good team for the dying older person and their family.

On the one hand, specialized palliative care places great emphasis on teamwork, which is often well integrated into that form of care. On the other hand, a limited team-based working method with an emphasis on single-point efforts on the part of the nurse has been reported. The NAs’ description of teamwork in the present study reveals that they are on the team’s fringes and cannot identify the role/function of other professional groups caring for the dying older person [36]. The development of multi-professional collaboration within general palliative care could increase nursing personnel’s opportunity to exchange knowledge, which would contribute to equal palliative care and raise the quality of end-of-life palliative home care for the older person at home. If the NAs perform most palliative care for older people dying at home, they need further education and training.

That the collaboration with RNs mostly took place via telephone and that the RNs had a different workplace hindered the building of relationships between NAs and RNs. The NAs expressed that the communication was sometimes insufficient because the RNs did not know the patients, which sometimes led to insufficient information about necessary care and, consequently, to the suffering of older persons. Another Swedish study has shown that older persons also experienced that the communication between the RN and the NA affected care [36]. That the RNs in this municipality work consultatively and are not involved in direct clinical care can be a decisive factor in the perceived distance in the descriptions of the collaboration in the team. The results might have been different had the NAs and the RNs worked closer to each other in the same organization.

NAs working in palliative care must receive sufficient support from the organization to handle the emotions that may arise [37]. The National Board of Health and Welfare has stated in the national guidelines [9] that the organization must offer ongoing training to the personnel to provide good palliative care. Otherwise, it might be difficult for the personnel to meet the patients’ palliative care needs, such as symptom relief and interaction with patients and their next of kin. In this study, the NAs were left alone with the feelings induced by their role as home carers of a dying older person.

The NAs interviewed in the present study had long working experience. However, they had not participated in any training, specifically in palliative care. Therefore, it is not surprising that they primarily relied on their experience-based knowledge when caring for dying older persons, which was also found in the study by Holmberg et al. [26]. Most NAs do not have a higher academic education (it is not a requirement for NA certification); therefore, they must be provided with support and time to reflect, discuss, and evaluate their experiences [38,39]. In addition, the NAs in the present study reported that some colleagues lacked knowledge about palliative care or sufficient language skills. A lack of language skills and low competence in palliative care are significant risk factors for dying older adults and lead to inferior quality of care [9].

The provided end-of-life palliative home care relies mainly on the experience and competence of the individual NA, which implies that the quality of care for older dying people has become dependent on individual factors rather than on professional ones. The varied competency between individual NAs jeopardizes patient safety in municipal care because it leads to uncertainty regarding subjective assessments and interpretations of older adults’ symptoms [38].

The findings in this study align with other studies that show a need for improvement in delivering good palliative care [11–17, 39] providing continuous training and practical guidance for NAs together with RNs could improve care quality. Such training and guidance could, for example, concern factors such as ensuring patients undergo an oral assessment, assessing pain, offering an end-of-life conversation, and how to hold post-bereavement meetings. Education about how to fulfil the four cornerstones must be organized to increase insight and knowledge to deliver good palliative care. It is crucial to have supportive management and organization to provide an education that achieves lasting positive effects [38, 41].

Further research should focus on intervention studies with education and training to ensure that the competence-enhancing efforts affect the palliative care of the dying older person. Furthermore, person-centered care and teamwork to improve palliative care must be investigated.

### Strength and limitations

The study’s low response rate might be seen as a major limitation and threatens external validity.

However, we believe that the data given by the NAs were of substantial content.

We invited NAs from six units, but only 14 NAs from two units responded. The response rate may have been influenced by the different information about the study provided by managers to the NAs. Moreover, the study was performed during the pandemic, and all healthcare providers around the globe had a hectic time. Therefore, it might have been busier at the other four units; thus, the study was not prioritized. Nevertheless, we included the 14 NAs that responded, and the sample was estimated to be sufficient to reach data saturation because we have NAs with different ages and working experience, and after ten interviews no new information came up.

We developed a semi-structured interview guide with open questions to answer the study’s aim and to ensure that the four cornerstones were included. The advantage of a semi-structured interview includes, for example, preparing questions beforehand to help guide the conversation and to keep respondents on topic, allowing for open-ended responses from participants for more in-depth information, and encouraging two-way communication. However, there are several limitations, including the challenge of finding an interviewer with the right amount of training to conduct the interview properly and the possibility of writing leading questions that can bias the interview. To limit potential bias in the study, the two authors, JB and MN, conducted the first ten interviews, while the remaining four were conducted with one author, either JB or MN. Additionally, MAG checked the guide to ensure there were no leading questions.

To reduce the risk of subjectivity [21], all four authors reflected on the content analysis results to guarantee trustworthiness [22]. In addition, quotations are presented to enable transparency in the analysis and to underline the study’s credibility [22]. A strength of the study is that all the NAs expressed the same wish, namely that palliative care in the older person’s home involves doing everything possible for the dying older person.

## Conclusion

In this study, the NAs’ experiences of providing end-of-life palliative home care were permeated by their desire to do everything possible to provide the best possible care based on organizational conditions that were not always favorable. They relied primarily on their experience when caring for the dying older person and expressed vulnerability.

Experience-based knowledge – developed in clinical situations as a combination of tacit knowledge, practical wisdom, intuition, and experience – is essential in healthcare. However, it should be seen as a complement to scientific knowledge. Therefore, there is a need for increased support from RNs as nursing leaders and an organization that offers training and supervision in palliative care to create good conditions for the NAs to perform safe, equal, high-quality end-of-life palliative home care for older people.

### Electronic supplementary material

Below is the link to the electronic supplementary material.

Supplementary Material 1

## Data Availability

Data is available on request from author 2 Josefine Brenner and author 3 Malin Nordberg.

## Declarations

This study followed the Swedish Ethical Review Act [40] and the Declaration of Helsinki: [41] NAs gave written informed consent after receiving written and verbal information about the study, including its purpose and the procedures involved. The NAs were informed that they could withdraw their participation at any time and that data were handled confidentially. The methods followed guidelines and regulations, including the institutional ethics code [8]. The Ethics Council at Malmö University assessed the project (HT21/Löpnr 9). Attention was given to the recruitment procedure to balance the ethical principles of autonomy, beneficence, and non-maleficence.

## References

[1] United Nations. World Population Prospects 2022. https://www.un.org/development/desa/pd/sites/www.un.org.development.desa.pd/files/wpp2022_summary_of_results.pdf

[2] World Health Organisation. Palliative care, fact sheets 2020. https://www.who.int/news-room/fact-sheets/detail/palliative-care.

[3] The share of the elderly population in Sweden aged 65 years or more in selected years from 1970 to 2022. Statista; 2022.

[4] Wilson DM, Cohen J, Deliens L et al. The preferred place of last days: results of a representative population-based public survey. J Palliat Med. 2013; 16(5): 502–508, http://www.ncbi.nlm.nih.gov/pubmed/23421538 (accessed May 31, 2023). 10.1089/jpm.2012.0262 23421538

[5] Vidal M, Rodriguez-Nunez A, Hui D et al. Place-of-death preferences among patients with cancer and family caregivers in inpatient and outpatient palliative care. BMJ Support Palliative Care. 2020. Available at: https://pubmed.ncbi.nlm.nih.gov/32253348/. Accessed May 31, 2023. 10.1136/bmjspcare-2019-002019 32253348

[6] Hoare S, Kelly MP, Barclay S (2019). Home care and end-of-life hospital admissions: a retrospective interview study in English primary and secondary care. Br J Gen Pract.

[7] Kogan AC, Li O, Fields T, Mosqueda L, Lorenz K (2022). Frontline provider perceptions of implementing home-based palliative care covered by an insurer. Health Service Res.

[8] Saarelainen SM, Vähäkangas A, Anttonen M. Religions. 2020;11(335). 10.3390/rel11070336.

[9] The National Board of Health and Welfare (2013). National knowledge support for good palliative care in the end of life: Guidance, recommendations and indicators. Support for steering and management.

[10] Mikaelsson Midlöv E, Lindberg T (2020). District nurses’ experiences of providing palliative care in the home: an interview study. Nordic J Nurs Res.

[11] Ye Z, Jing L, Zhang H, Qin Y, Chen H, Yang J, Zhu R, Wang J, Zhang H, Xu Y, Chu Y (2023). Attitudes and influencing factors of nursing assistants towards hospice and palliative care nursing in Chinese nursing homes: a cross sectional study. BMC Palliat Care.

[12] Sundström M, Blomqvist K, Edberg A-K, Rämgård M (2019). The context of care matters: older people’s existential loneliness from the perspective of healthcare professionals—A multiple case study. Int J Older People Nurs.

[13] Craftman ÅG, Pakpour AH, Calderon H, Meling A, Browall M, Hagelin CL (2022). Home care assistants’ attitudes and perceptions of caring for people at the end-of-life in their homes in Sweden. Health Soc Care Community.

[14] Beck I, Törnquist A, Broström L, Edberg A-K (2012). Having to focus on doing rather than being—nurse assistants’ experience of palliative care in municipal residential care settings. Int J Nurs Stud.

[15] Fee A, Muldrew D, Slater P, Payne S, Mcllfatrick S, McConell T, Finlay D-A, Hasson F (2020). The roles, responsibilities and practices of healthcare assistants in out-of-hours community palliative care: a systematic scoping review. Palliat Med.

[16] Erel M, Marcus EL, Dekeyser-Ganz F (2017). Barriers to palliative care for advanced dementia: a scoping review. Ann Palliat Med.

[17] Udo C, Neljesjö M, Strömkvist I, Elf M. A qualitative study of assistant nurses’ experiences of palliative care in residential care. Nurs Open 2018, 5:527–35. 10.1002/nop2.159. 10.1002/nop2.159 PMC6177554 30338098

[18] The Health and Medical, Services Act SOU. 2017: 30. Coordinated development for good quality, local healthcare. The Swedish government. https://www.government.se/49f223/contentassets/7917e61ab62c421d8d007174d6df769c/good-quality-local-health-care--a-joint-roadmap-and-vision-summary-sou2017-53.pdf.

[19] Morgan DG, Kosteniuk JG, O’Connell ME, Bello-Haas VD, Stewart NJ, Karunanayake C (2016). Dementia-related work activities of home care nurses and aides: frequency, perceived competence, and continuing education priorities. Educ Gerontol.

[20] Wallerstedt B, Andershed B, Benzein E (2014). Family members’ caregiving situations in palliative home care when sitting service is received: the understanding of multiple realities. Palliat Support Care.

[21] Polit DF, Beck CT. (2020) Nursing research. Generating and assessing evidence for nursing practice. Edition 11. Philadelphia, Lippincott Williams & Wilkins.

[22] Burnard P, Gill P, Stewart K, Treasure E, Chadwick B. Analysing and presenting qualitative data. Br Dent J. 2008;204 (8). 10.1038/sj.bdj.2008.292 18438371

[23] CEQUA, LTC network, 2017, Sweden Country Report. Quality and cost-effectiveness in long-term care and dependency prevention. https://aldrecentrum.se/wp-content/uploads/2020/07/quality_and_cost-effectiveness_in_long-term_care_and_d.pdf.

[24] The Health and Medical, Services Act. SOU 2020:80. The elderly care in the pandemic. The Swedish government. https://www.government.se/legal-documents/2020/12/summary-of-sou-202080-elderly-care-during-the-pandemic/.

[25] Gomes B, Calanzani N, Gysels M (2013). Heterogeneity and changes in preferences for dying at home: a systematic review. BMC Palliat Care.

[26] Holmberg B, Hellström I, Österlind J (2019). End-of-life care in a nursing home: Assistant nurses’ perspectives. Nurs Ethics.

[27] Åvik Persson H, Sandgren A, Fürst C-J, Ahlström G, Behm L. Early and late signs that precede dying among older persons in nursing homes: the multidisciplinary team’s perspective. BMC Geriatr. 2018; (1), 1–11. 10.1186/s12877-018-0825-0 PMC6000966 29898674

[28] Voumard R, Truchard R, Benaroyo L, Borasio D, Bula C, Jox J. Geriatric palliative care: a view of its concept, challenges and strategies. BMC Geriatr, 2018;18 (220). 10.1186/s12877-018-0914-0 PMC6148954 30236063

[29] Bollig G, Gjengedal E, Rosland JH. They know! – do they? A qualitative study of residents and relatives’ views on advance care planning, end-of-life care, and decision-making in nursing homes. 2016;30 (5), 456–70. 10.1177/0269216315605753 PMC4838176 26396227

[30] Österlind J, Hansebo G, Andersson J, Ternestedt B-M, Hellström I (2011). A discourse of silence: Professional carers reasoning about death and dying in nursing homes. Ageing Soc.

[31] Eriksson G, Bergstedt T, Melin-Johansson C. The need for palliative care education, support, and reflection among rural nurses and other staff: a quantitative study. Palliat Supportive Care 2015;13 (2). 10.1017/S1478951513001272 24576441

[32] Beck I, Pålsson C, Bengtsson Tops A. (2018) Upholding an ideal image of palliative work in the face of obstacles. *International Journal of Palliative Nursing* 2018;24 (12). 10.12968/ijpn.2018.24.12.611 30571249

[33] Abrams R, Vandrevala T, Samsi K, Manthorpe J (2019). The need for flexibility when negotiating professional boundaries in the context of home care, dementia and end-of-life. Ageing Soc.

[34] Jarling A, Rydström I, Ernsth Bravell M, Nyström M (2020). Dalheim- Englund A-C.Perceptions of professional responsibility when caring for older people in Home Care in Sweden. J Commun Health Nurs.

[35] Skorpen Tarberg A, Kvangarsnes M, Hole T, Thronæs M, Støve Madssen T, Landstad BJ (2019). Silent voices: family caregivers’ narratives of involvement in palliative care. Nurs Open.

[36] Claesson M, Josefsson K, Jonasson L (2021). My registered nurse: older people’s experiences of registered nurses’ leadership close to them in community home care in Sweden. Int J Older People Nurs.

[37] Cronfalk B, Ternestedt B-M, Franklin Larsson L-L, Henriksen E, Norberg A, Österlind J. (2015) Utilization of palliative care principles in nursing home care: *Educational interventions. Palliative & Supportive Care* 2015;13 (6), 1745-1753. 39 Fryer S, Bellamy G, Morgan T, Gott M. Sometimes I’ve gone home feeling that my voice hasn’t been heard: a focus group study exploring the views and experiences of healthcare assistants when caring for dying residents. *BMC Palliative Care*, 2016;15, 1–9.

[38] Schildmeijer K, Wallerstedt B, Ekstedt M. (2019) Healthcare Professionals’ Perceptions of Risk When Care Is Given in Patients’ Homes. *Home Healthcare Now*. 2019;37 (2), 97-105. 41 Beck I, Törnquist A, Edberg A-K. Nurse assistants’ experience of an intervention focused on a palliative care approach for older people in residential care. *International journal of people nursing*, 2014;9(2), 40–50.

[39] Bolt SR, Meijer JMM, van der Steen JT, Schols JM, Zwakhalen SMG (2020). Nursing staff needs in providing Palliative Care for persons with dementia at Home or in nursing homes: a Survey. J Nurs Scholarsh.

[40] The Swedish ethical review act. SFS, 2003:460 https://www.onep.se/media/2348/the_ethical_review_act.pdf.

[41] World Medical Association (2013). World Medical Association Declaration of Helsinki: ethical principles for Medical Research Involving human subjects. JAMA.

