# Supplementary material for: Nurse assistants’ perception of caring for older persons who are dying in their own home: An interview study
Source: BMC Palliat Care. 2024 Mar 12;23:70. doi: 10.1186/s12904-024-01399-2 (PMC10929109; doi:10.1186/s12904-024-01399-2)
Supplement: Supplementary file 1 — Supplementary Material 1 [file 12904_2024_1399_MOESM1_ESM.docx]

INTERVIEW GUIDE

Background questions:

Age.

Sex.

How long have you worked in home care?

What is your education? (including other education except healthcare)

What have you learned that you believe you can use in palliative care today?

Or (if no training): What previous experiences do you have of caring for dying people that you can use in your work today?

Questions

Tell us about your experience of end-of-life care at home? (Can you elaborate further; can you give examples?)

Based on your work in home care, What is palliative care at the end of life in your perception? What does it mean? What could that mean?

(Clarify what we mean with care at the end of life)

What does it mean to you to alleviate symptoms in the palliative older person? What symptoms did you experience that they have? What are you doing then? What can others help with?

How do you detect these symptoms in the older person at the end of life?

How do you experience the collaboration with people from other professions, such as nurses, doctors, rehab staff, your boss? (How?)

Can you explain to me how contact with other people like a nurse, doctor, rehab might look like?

Can you describe how contact with relatives might look like when you have cared for a dying older person? What was the contact about? How did you experience that contact?

Can you tell us about a situation that was experienced to be valuable (that felt good, or meant something) when you cared for an older person at the end of life at home? (Can you elaborate a bit; can you give examples?)

Tell me if you think there are any difficulties for you as a nursing assistant in the home service to provide palliative care at a person’s home? (Can you elaborate on it; can you give examples?)

Do you have experience as a nursing assistant in the home service of being by the older person’s side when they died? How did you experience that situation?

How do you experience your role as nursing assistant in the home service caring for a person you may have known for a long time? How is the relationship affected at the end of life? Is it different to care for a palliative care person with whom you have had a relationship before?

Is there anything that you think could be improved in palliative care at home? (Can you give some suggestions on how?)

Is there anything else you'd like to add before we wrap up?

Supplementary questions:

Can you give examples? Can you tell me more? What did you think then? How did you experience it? How did you handle it? How did you go about it then?
